# Supplementary material for: Inhibitory Effects against Alpha-Amylase of an Enriched Polyphenol Extract from Pericarp of Mangosteen (Garcinia mangostana)
Source: Foods. 2022 Mar 29;11(7):1001. doi: 10.3390/foods11071001 (PMC8997748; doi:10.3390/foods11071001)
Supplement: Supplementary file 1 [file foods-11-01001-s001.zip › foods-1651706-supplementary.pdf]

Supplementary Materials

Table S1. Factors and levels chosen for the orthogonal array experiment for MPPs extraction.

**factors**	**level**		
	**low(1)**	**medium(2)**	**high(3)**
A. solid to solvent ratio(mg/mL)	1:40	1:45	1:50
B. pH	1.0	1.5	2.0
C. temperature/ (℃)	60	70	80
D. time/(h)	1.0	1.5	2.0

Table S2. Orthogonal test results of extraction yield of MPPs

number	mangosteen pericarp powder/g	solid to solvent ratio(A)	pH(B)	temperature(C)	time(D)	extraction yield/%
1	3.00	1	1	1	1	2.65± 0.07
2	3.00	1	2	2	2	2.77 ± 0.09
3	3.00	1	3	3	3	2.98 ± 0.05
4	3.00	2	1	2	3	2.91 ± 0.04
5	3.00	2	2	3	1	3.07 ± 0.08
6	3.00	2	3	1	2	2.85 ± 0.05
7	3.00	3	1	3	2	3.08 ± 0.07
8	3.00	3	2	1	3	2.99 ± 0.06
9	3.00	3	3	2	1	3.09 ± 0.08
K_1		8.40	8.64	8.49	8.81	
K_2		8.83	8.83	8.77	8.70	
K_3		9.16	8.92	9.13	8.88	
k_1		2.80	2.88	2.83	2.94	
k_2		2.94	2.94	2.92	2.90	
k_3		3.05	2.97	3.04	2.96	
R		0.25	0.09	0.21	0.06	
order				A>C>B>D		
optimum levels		A_3	B_3	C_3	D_3	
optimum factors				$A_3B_3C_3D_3$		

Table S3. Binding energy between α-amylase with representative compounds in MPPs calculated by Autodock

compound	binding energy(kcal/mol)	RMSD(Å)
Garcimangosone D	-4.57	2.290
β-mangostin	-4.50	0.176
Garcinone D	-4.47	0.036
Cyanidin-3-*O*-glucoside	-3.96	1.919
Pelargonidin-3-*O*-glucoside	-4.85	0.755
Proanthocyanidin A2	-4.92	0.001
(E)C	-4.90	0.001
